# Practical judgment in aging: examining behavioral vulnerabilities and neurobiological correlates

**DOI:** 10.3389/fpsyg.2026.1709372

**Published:** 2026-03-03

**Authors:** Nicole Sergeyev, Abi Heller-Wight, Caroline Nester Rooney, Nadia Paré, Anjali Krishnan, David G. Ellis, Chloe Moffitt, Robert M. Roth, David E. Warren, Laura Rabin

**Affiliations:** 1 Department of Psychology and Neuroscience, Duke University, Durham, NC, United States; 2 Department of Psychology, Brooklyn College of The City University of New York, Brooklyn, NY, United States; 3 Department of Neurological Sciences, University of Nebraska Medical Center, Omaha, NE, United States; 4 Department of Psychology, The Graduate Center of The City University of New York, New York, NY, United States; 5 Department of Psychiatry and Human Behavior, Brown University, Providence, RI, United States; 6 Department of Psychiatry, UCONN Health, Farmington, CT, United States; 7 Department of Neurosurgery, University of Nebraska Medical Center, Omaha, NE, United States; 8 Department of Neurology, Albert Einstein College of Medicine, Bronx, NY, United States; 9 Department of Psychiatry, Dartmouth Geisel School of Medicine, Lebanon, NH, United States

**Keywords:** decision-making, practical judgment, scam susceptibility, structural connectivity, white matter changes

## Abstract

**Background:**

Real-world decision-making often relies on practical judgment—the ability to evaluate information, anticipate consequences, and respond in an adaptive manner. Despite its importance for preserving independence in aging, this construct is understudied in older adults without dementia. Moreover, there is a gap in knowledge about the association of objective and informant-rated measures of judgment and other variables that impact everyday functioning such as scam vulnerability and white matter integrity, given that structural white matter changes may precede objective cognitive decline.

**Methods:**

Community-dwelling older adults classified as cognitively unimpaired (CU; *n* = 30, Mage = 73.57 ± 1.22 years), or as having subjective cognitive decline (SCD; *n* = 33, Mage = 72.49 ± 1.13 years) or mild cognitive impairment (MCI; *n* = 30, Mage = 78.43 ± 1.53 years) completed the Test of Practical Judgment (TOP-J), an ecologically useful measure of everyday judgment, along with a self-report measure of scam susceptibility (Susceptibility to Scams). Informants provided ratings of participants’ practical judgment abilities (TOP-J Informant) and vulnerability to exploitation with the Social Vulnerability Scale and a binary item measuring scam engagement. Kruskal-Wallis and Spearman correlation tests were used to examine group differences and associations between practical judgment and measures of vulnerability. Participants underwent structural MRI; diffusion imaging data were used to extract fractional anisotropy (FA) values and exploratory region-to-region connectivity metrics for the uncinate fasciculus (UF). Associations between UF integrity and structural connectivity with judgment were examined in the overall sample using regression analyses controlling for age, and group differences in white matter integrity were examined using ANCOVA.

**Results:**

Greater structural connectivity of the UF in right temporal-frontal and frontal–frontal regions was significantly associated with better informant-reported judgment in the overall sample. Better objective and informant-based practical judgment scores were significantly associated with lower informant-reported scam engagement. Objective and informant-rated judgment abilities were significantly worse among participants with MCI compared to CU.

**Conclusion:**

Findings highlight the value of using objective and informant sources to capture real-world judgment ability, with implications for the prevention of harmful outcomes. Our results also suggest that structural connectivity within the UF may be a promising biomarker of impaired judgment in older adults without dementia.

## Introduction

Decision-making is a multi-step process that engages higher-order cognitive resources, allowing individuals to turn deliberate thought into calculated action (Vohs and Luce, 2019). A key component of the decision-making process is judgment, that is, the ability to perceive relevant information, evaluate potential outcomes, and draw informed conclusions about a situation (Guayara-Quinn et al., 2021; Rabin et al., 2007). This evaluative process and behavioral follow-through rely on the integration of executive functions, socioemotional skills, and prior experience (Rabin et al., 2007). Although actions can occur without sound judgment, thoughtfully evaluating a situation can enhance the likelihood of achieving a preferred outcome (Capucho and Brucki, 2011). In daily life, practical judgment involves anticipating consequences, weighing options, and recognizing risks—skills that are essential not only in complex decision-making, but also in routine activities such as managing medications, driving, or budgeting (Stern and Carstensen, 2000).

Cognitive abilities that contribute to judgment, such as memory, processing speed, and cognitive flexibility, are supported by distributed networks of brain regions including portions of the association cortex in the frontal and temporal lobes (Kochunov et al., 2010; Staffaroni et al., 2018). These networks, and the cognitive abilities they support, exhibit normative age-related declines (Cadar et al., 2018; Idowu and Szameitat, 2023; Löckenhoff and Rutt, 2015; Murman, 2015). Specifically, changes in judgment have been linked to changes in structural connectivity within key networks as measured by integrity of white matter and myelinated tracts, particularly in frontal and temporal association regions (Brancucci, 2012; Metzler-Baddeley et al., 2011; Di Plinio et al., 2025). Thus, age-related changes in judgment are often associated with targeted network changes in brain structure or function, and conversely selective changes in brain structure and function may alter judgment abilities.

Subtle declines in judgment observed in healthy aging can be exacerbated by neurodegenerative disease processes and their associated pathologies. As the most common neurodegenerative disease causing dementia, Alzheimer’s disease (AD) and its prodromes merit special scrutiny in the domain of judgment. Progression along the cognitive continuum from cognitively unimpaired toward dementia, notably in prodromal AD stages, can also significantly impair judgment abilities. These include subjective cognitive decline (SCD), characterized by self-experienced persistent cognitive decline in the absence of measurable neuropsychological impairment, and mild cognitive impairment (MCI), in which objective cognitive impairment and mild functional impairment are present, yet do not meet criteria for dementia (Snyder et al., 2011; Knopman and Petersen, 2014). Older adults without dementia who experience subtle changes in their cognition may run into challenges in their everyday functioning and be susceptible to poor decision-making (Boyle et al., 2012; Perneczky et al., 2006; Makino et al., 2020; Mangin et al., 2023). These age-related changes in cognitive abilities that support judgment can impair the ability of older adults to make adaptive decisions in high-pressure situations, such as choosing between medical treatment options or recognizing, and further, avoiding scams (Okonkwo et al., 2007; Coundouris et al., 2023).

There is limited research examining how judgment relates to real-life outcomes that are directly tied to safety in older adults, such as vulnerability to fraud or functional independence (Quinn et al., 2018; Mansbach et al., 2019; Mayo et al., 2013). Understanding this association is especially urgent given growing evidence that community-dwelling older adults are increasingly targeted by, and falling for, scam attempts (Burnes et al., 2017; Yu et al., 2021; Yu et al., 2022), which may be a key manifestation of compromised judgment abilities. Between 2021 and 2023, elder fraud victimization increased by 209%, while reported financial losses rose by 419% (Federal Bureau of Investigation, 2023), primarily due to a surge in scams related to customer support and investment-related schemes. Prior studies linking cognition to scam vulnerability have primarily focused on core executive abilities (Judges et al., 2017; Han et al., 2016) or isolated measures of financial and health decision-making (Yu et al., 2021), without including formal tests of practical judgment. Such studies often rely on brief scam susceptibility scales that may not capture the threats individuals confront in their daily lives (Kapasi et al., 2021; James et al., 2014). Further, no study to date has assessed both objective and informant-based measures of judgment in relation to scam vulnerability—potentially overlooking early indicators of risk, as close friends or family may provide a richer understanding of subtle, day-to-day declines in judgment (Rabin et al., 2022). These gaps in the literature leave open questions regarding associations between judgment and scam vulnerability in older adults.

Cognitive decline has emerged as a significant risk factor for scam susceptibility, as people with MCI have been shown to be more susceptible to telephone fraud (Judges et al., 2017); moreover, both older age and lower working memory have been associated with vulnerability to so-called “phishing” attacks (Pehlivanoglu et al., 2024; Han et al., 2016). Older adults with early signs of objective cognitive impairment show significant declines in their financial decision-making capacity (Lichtenberg et al., 2024; Triebel et al., 2009) and reduced decision-making performance in risky or ambiguous situations, (Sun et al., 2020) potentially increasing their vulnerability to exploitation, as compared to their healthy counterparts. While MCI is associated with greater vulnerability to scams, individuals with more severe cognitive impairment may demonstrate lower risk due to reduced exposure and engagement (Ueno et al., 2021). These findings would be consistent with an inverted U-shaped curve of scam vulnerability, where cognitively normal older adults may be less vulnerable due to relatively preserved judgment, and individuals with moderate-to-severe dementia may show reduced vulnerability due to limited independence and exposure. In contrast, independent older adults with SCD or MCI may face greater vulnerability to scams due to a combination of impaired judgment and typical exposure. If true, older adults with SCD or MCI may represent a particularly high-risk group, indicating the need for further research into the relationship between judgment and real-world fraud vulnerability in preclinical populations.

Uncovering the neurobiological correlates of practical judgment in older adults may also be useful to identify those at heightened risk for impaired decision-making. Cortical white matter (WM) integrity has been associated with financial decision-making capacity (Sunderaraman et al., 2022) and has been shown to differentiate between healthy older adults with strong versus poor decision-making skills (Timpe et al., 2011). The uncinate fasciculus (UF) is a major white-matter projection connecting key decision-making regions, particularly the orbitofrontal cortex and the anterior temporal association cortex (Wallis, 2007). While the exact functional role of the UF remains unclear, studies have linked the UF to the integration of episodic memory, language, and socio-emotional processing in clinical and non-clinical populations across the lifespan (Von Der Heide et al., 2013; Granger et al., 2021), and the UF has also been implicated in social conceptual processing in patients with frontotemporal dementia (Strikwerda-Brown et al., 2021). According to Von der Heide et al. (2013) the integration of memory and socio-emotional information makes this tract critical for forming associations between stimuli and rewards or punishments, ultimately contributing to decision-making. In addition, the deterioration of the UF has been discussed as a possible neural marker of MCI (Kiuchi et al., 2009) and is predictive of conversion to AD (Hiyoshi-Taniguchi et al., 2015; Larroza et al., 2014). Thus, examining associations between judgment ability and the microstructural integrity of the UF may offer unique insight into the differences in brain systems that underlie impaired judgment in aging, whether healthy or pathological.

The present study has two primary aims: (1) to examine associations between practical judgment, measured via both a validated, ecologically-relevant standardized test and informant-report measures tapping the same abilities, and vulnerability to exploitation; and (2) to investigate whether white matter differences, particularly in the UF, is predictive of judgment ability in older adults with varying levels of cognitive concerns and impairment. We hypothesized that: (1) poorer objective and informant-reported judgment would be associated with greater scam susceptibility and social vulnerability; (2) poorer judgment scores, both objective and informant-reported, would be associated with the WM differences in the UF; and (3) cognitive impairment would have a meaningful impact on outcomes of interest, with individuals with MCI expected to perform worse on measures of judgment while also having greater self- or informant-reported vulnerability to scams.

## Materials and methods

### Participants and procedures

Participants were recruited from the University of Nebraska Medical Center (UNMC) as well as surrounding assisted living facilities, community centers, and religious organizations in Omaha, NE. Approximately 15–20% of the participants were recruited from UNMC (these individuals included clinic patients and their spouses/peers), and the remainder were recruited from the community. Inclusion criteria required participants to be at least 60 years of age, capable of providing written informed consent, and to have an informant who had at least weekly contact with them for a minimum of 2 years. Individuals were excluded if there was a previous diagnosis of neurological disease other than MCI (e.g., focal stroke detected on imaging, Parkinson’s disease, epilepsy, multiple sclerosis), history of head trauma with loss of consciousness greater than 5 min, neurodevelopmental disorder, prior diagnosis of learning disability, substance use disorder, and psychiatric disorder. In addition, we excluded left-handed individuals due to the project’s neuroimaging component, as well as participants with chronic use of medications that could affect cognition, such as anticholinergic medications (including sleep aids with diphenhydramine) and opioids.

All participants provided informed written consent according to UNMC Institutional Review Board (IRB) guidelines. Informants were spouses, family members, friends, and caregivers of participants who provided information about areas of participant cognition and function, via clinical interview and informant rating scales. Participants underwent neuropsychological testing including measures of global cognition, memory, attention, executive functions, language, mood, and functional status/capacity. These evaluations were conducted by trained research assistants under the supervision of licensed clinical psychologists/neuropsychologists. Participation was remunerated on a prorated basis of $15/h.

### Diagnostic classification

Diagnostic classification of each participant was established through case reviews made by three neuropsychologists based on commonly applied criteria including: Petersen et al. (1999), Winblad et al. (2004), DSM-5 (American Psychiatric Association, 2013), and the syndromal cognitive staging scheme proposed by Jack et al. (2018), which is applicable to research cohorts independent from biomarker profile. Participants were categorized into three groups: cognitively unimpaired (CU), subjective cognitive decline (SCD), or mild cognitive impairment (MCI), based on clinical judgment and demographic-adjusted scores on a battery of standardized cognitive tests used to screen for and classify MCI or dementia.

Participants were categorized as CU if: (1) their scores were within normal limits on a battery of standardized cognitive tests; (2) there was no report of significant cognitive concerns based on the clinical interview with the study’s psychologists and on the self and informant versions of the Brief Informant Report of Neurobehavioral Symptomology (BINS; Paré et al., 2020); and (3) scores on the Amsterdam Activities of Daily Living Questionnaire—Short Version (A-IADL-Q-SV; Jutten et al., 2017), a validated measure of functional status (Dubbelman et al., 2022) fell in the “no impairment” range. Participants were categorized as SCD (Jessen et al., 2014) if: (1) there was self-reported persistent cognitive decline from a prior level of cognitive functioning that was independent of an acute event, as assessed during the clinical interview with the study’s licensed clinical psychologist(s) and through BINS responses, which showed an elevated level of concern by the participant and/or informant; (2) the individual’s scores were within normal limits on a battery of standardized cognitive tests; and (3) scores fell in the “no impairment” range on the A-IADL-Q-SV. Participants were classified as MCI if: (1) scores on at least two memory tests were at least 1.5 SD below the normative age- and education mean; (2) there was a reported decline in cognitive performance from the participant or study informant on the BINS; and (3) scores fell between the “no impairment” and “mild impairment ranges” on the A-IADL-Q-SV.

### MRI data acquisition

Participants underwent MRI with a Siemens 3 T Prisma scanner, with protocols adapted from the Human Connectome Project Development/Aging Protocol (Harms et al., 2018). All MRI data were collected from participants in Omaha, Nebraska, at the UNMC Core for Advanced Magnetic Resonance Imaging (CAMRI, RRID: SCR_022469). After completing the safety screening, participants completed a brain MRI scan with a 32-channel head coil. T1-weighted whole-brain images were acquired with 0.8 mm isotropic resolution, TR = 2.4 s, and TE = 2.22 ms. Diffusion imaging data were acquired with 1.5 mm isotropic voxel size, TR = 3.23 s, TE = 89.2 ms, b-value shells of 1,500 and 3,000 s/mm^2^, and 185 diffusion-weighted directions per shell. Additionally, reverse phase-encoding spin echo images were acquired for susceptibility distortion correction of the diffusion images.

### Diffusion image processing

**Figure 1:**
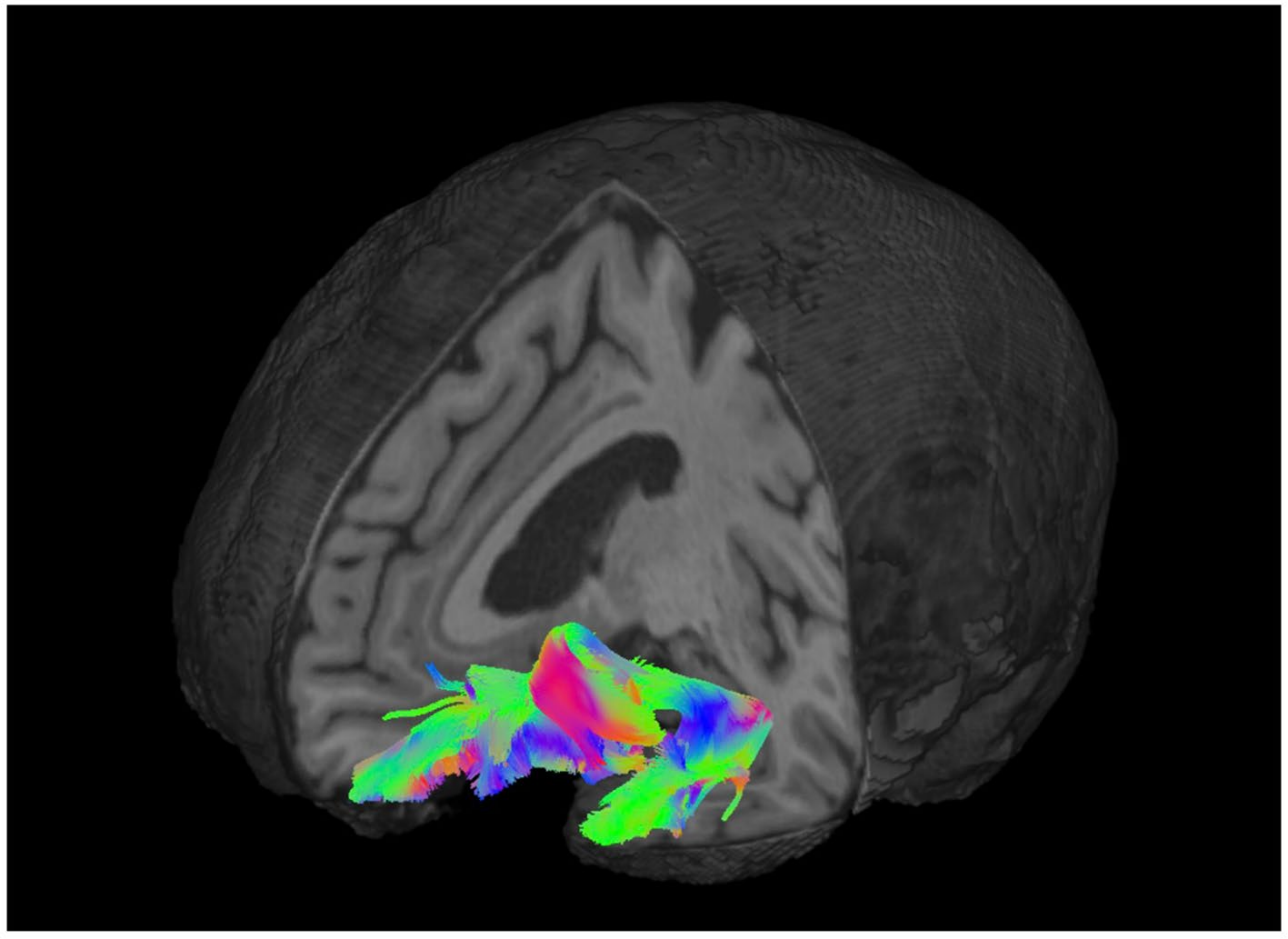
Tractography of the left uncinate fasciculus (UF). Three-dimensional representational reconstruction of the left UF overlaid on a T1-weighted anatomical image. Fiber pathways are color-coded by white matter diffusion direction (red = left–right, green = anterior–posterior, blue = superior–inferior).

Diffusion-weighted images were preprocessed with QSIPrep software (Cieslak et al., 2021), which was used to perform denoising (Veraart et al., 2016), bias correction (Tustison et al., 2010), motion correction, eddy current correction (Andersson et al., 2003), susceptibility distortion correction (Andersson and Sotiropoulos, 2016), and T1-weighted skull stripping (Hoopes et al., 2022) implemented in Nipype (Esteban et al., 2020). QSIRecon and DSI Studio was then used to compute diffusion orientation distribution functions using generalized q-sampling imaging (Yeh et al., 2010), perform tract reconstruction and automatic tract classification (Yeh, 2020; Yeh, 2022) with DSI Studio as well as tensor fitting. Fractional anisotropy values were calculated for each participant using DSI Studio software. See Figure 1 for a visualization of the target tract, the UF.

**Figure 2:**
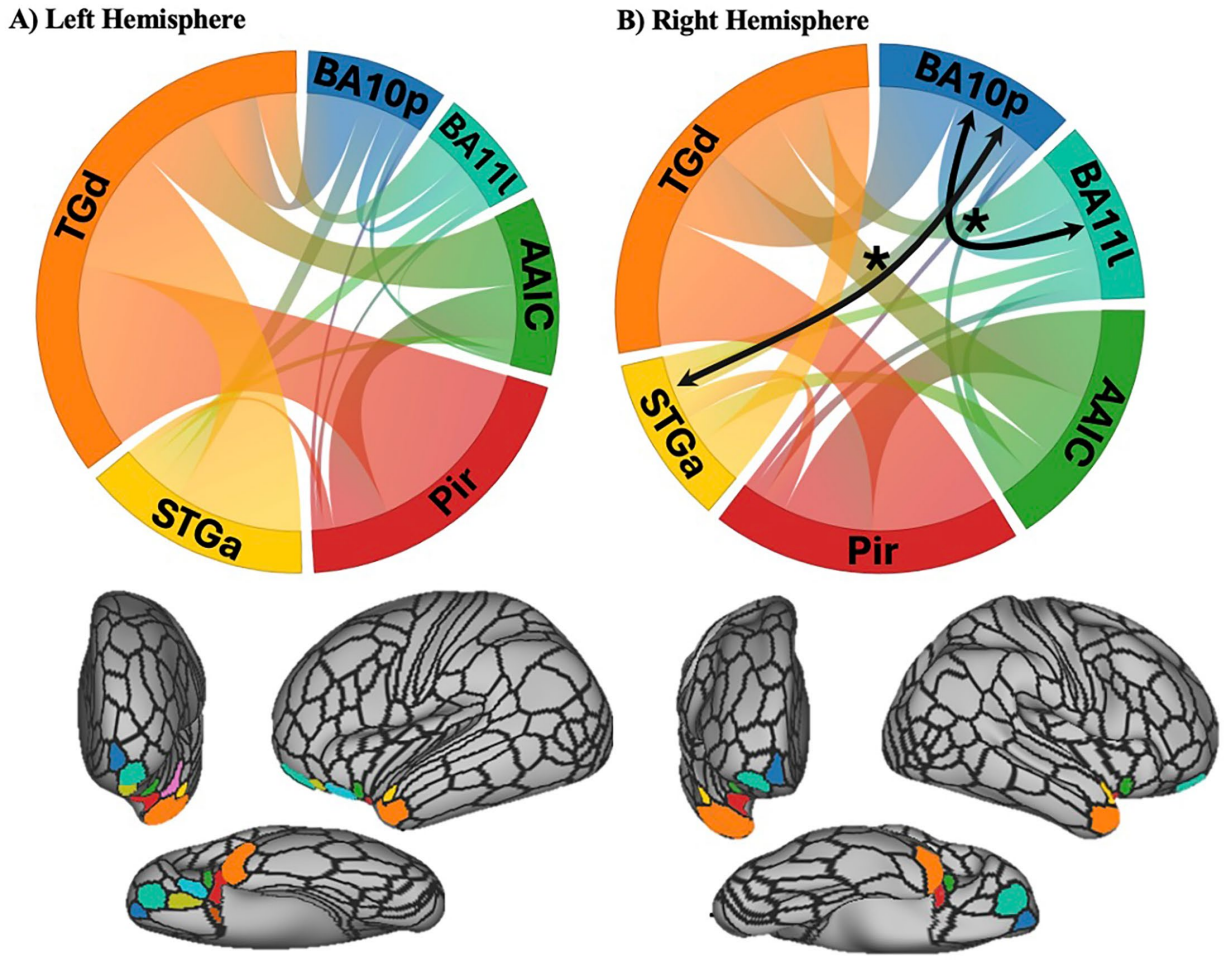
Structural connectivity of the uncinate fasciculus (UF) by hemisphere curved bands in the circular plots depict structural connections between cortical regions connected by the UF, with colors indicating the corresponding cortical regions. **(A)** Left hemisphere white matter connectivity and associated parcels shown from a frontal, lateral, and ventral view. No connections were associated with TOP-J informant in the left hemisphere. **(B)** Right hemisphere white matter connectivity and associated parcels from a frontal, lateral, and ventral view. Black arrows and asterisks denote connections significantly associated with TOP-J informant scores. TGd: area TG dorsal; BA10p: Broadmann area 10 polar; BA111: Broadmann area 11 lateral; AAIC: anterior agranular insula complex; Pir: piriform cortex; STGa: superior temporal gyrus anterior.

To further characterize associations between UF white matter structure and practical judgment, structural connectivity of the extracted uncinate fasciculus (UF) was used to assess connectivity between frontal and temporal regions in relation to TOP-J scores. The HCP-MMP parcellation (Glasser et al., 2016) was applied to construct a UF connectivity matrix, defined by the number of streamlines connecting each region of interest (ROI) pair (Yeh, 2020). A sparse matrix including only regions connected by the UF was created and streamline counts were normalized by dividing each ROI-to-ROI count by the total number of UF streamlines for that subject (Figure 2). Thus, each ROI-to-ROI value represented the proportion of all streamlines of the total UF tract connecting the two ROIs. Based on this proportional connection strength, the strongest (top 30%) of ROI-to-ROI connections were then analyzed in relation to TOP-J scores. This exploratory analysis allowed us to characterize the relationship between structural connectivity and judgment across the sample.

### Judgment measures

The Test of Practical Judgment^1^ (TOP-J; Guayara-Quinn et al., 2021; Rabin et al., 2007) is a widely used, well-validated measure of judgment and decision-making capacity in older adults across safety, medical, financial, and ethical domains. The measure consists of open-ended questions in which the participant is verbally presented with brief scenarios outlining everyday problems and is asked how they would respond. Responses to each item are recorded and scored on a 0–3-point scale with higher scores indicating better judgment for a total maximum score of 45. All responses were scored by a trained rater who was blinded to diagnostic group. The TOP-J has demonstrated strong psychometric support in the assessment of judgment of older adults (Guayara-Quinn et al., 2021). We used TOP-J Form A, which contains 15 items split between domains (4 safety, 3 medical, 3 financial, and 5 social/ethical) and which has key characteristics typical of robust, rigorous neuropsychological instruments (test–retest reliability of 0.86, inter-rater reliability of 0.96, and internal consistency of 0.68) (Rabin et al., 2007). The TOP-J has demonstrated sensitivity to subtle impairments in higher-order reasoning (Durant et al., 2018) by distinguishing individuals with MCI from those who are CU (Rabin et al., 2007).

The Test of Practical Judgment-Informant (TOP-J-Informant; Rabin et al., 2022) is a 15-item measure developed as an informant counterpart to the TOP-J to assess judgment across safety, medical, financial, and social situations. The item responses range from 0 = *normal ability/almost never a problem*; 1 = *mild difficulty/sometimes a problem*; 2 = *moderate difficulty/often a problem* to 3 = *severe difficulty/almost always a problem* with higher scores reflecting greater judgment difficulties. The total range of the measure is 0–45. The TOP-J-Informant has demonstrated reliability of 0.95 (Rabin et al., 2022) and Cronbach’s alpha for the current sample is 0.89.

### Scam vulnerability measures

Informants responded to a binary (yes = 0/no = 1) question targeting scam-susceptibility: “Since the beginning of the COVID-19 pandemic, has your loved one fallen prey or come close to falling prey to a scam?” and were given the option to provide additional information about the incident in an open-response field.

The Social Vulnerability Scale (SVS; Pinsker et al., 2011) is an informant-based measure used to assess vulnerability to financial exploitation in older adults. For each item, the informant is instructed to rate how often the participant has been subject to fraud, exploitation, and deception, with items scored from 0 = *never to* 4 = *always*. The total score is the sum of all the rated responses, with higher scores reflecting greater social susceptibility to abuse and exploitation. The SVS includes two subscales (Gullibility and Credulity) that serve as markers of exploitation, but in this study only the overall SVS score was employed.

The Susceptibility to Scams Scale (STS; Financial Industry Regulatory Meter, 2013; AARP, 1999) is a five-item self-report measure that measures risky financial decision-making and vulnerability to fraud (e.g., staying on the phone with a telemarketer, answering calls from unknown numbers). Item responses range from 0 = *strongly agree* to 7 = *strongly disagree*. The total score is the sum of the five items (0–35), with items 1, 2, and 5 reverse-coded so that higher scores reflect greater susceptibility. Items were adapted from official statements in the Financial Industry Regulatory Risk Meter (2013) and American Association of Retired Persons (AARP, 1999). The intraclass correlation coefficient for the measure was 0.63 in past research (James et al., 2014).

### Analyses

Descriptive statistics were calculated for demographic variables and a test of global functioning in the overall sample and by diagnostic groups (CU, SCD, and MCI). Open-ended responses (*n* = 6) describing real-world scam experiences were included as observations. A Shapiro–Wilk test was performed and showed that the distribution of total TOP-J (*W* = 0.957, *p* = 0.003) and TOP-J-I (*W* = 0.704, *p* < 0.001) scores departed significantly from normality. In addition, Levene’s test for equality of variances was found to be violated for the TOP-J-Informant, *F*(2, 90) = 21.453, *p* < 0.001, but not the TOP-J, *F*(2, 90) = 2.381, *p* = 0.098. As the assumptions for a one-factor analysis of variance were not met, we used the Kruskal-Wallis test to compare performance on the TOP-J across diagnostic groups, followed by pairwise comparisons. Spearman correlational analyses were conducted to determine the association between TOP-J scores and measures of scam susceptibility and social vulnerability.

Associations between judgment and white matter integrity of the UF were tested using multiple regression. Four separate linear regressions were conducted with FA values of the left and right uncinate fasciculus (FA_UF_) as predictors and TOP-J and TOP-J-Informant scores as dependent variables. In line with prior DTI research, chronological age was included as a covariate in regression analyses (Matijevic and Ryan, 2025; Behler et al., 2021). The UF was selected as the primary tract of interest based on existing empirical work. A Bonferroni correction for multiple comparisons was applied to the left and right UF analyses (adjusted *a* = 0.025).

We tested whether white matter integrity differed between groups. First, a Shapiro–Wilk test was performed and showed that the distribution of left (*W* = 0.980, *p* = 0.161) and right (*W* = 0.986, *p* = 0.449) FA_UF_ was normally distributed. Therefore, we used an ANCOVA to compare FA_UF_ across diagnostic groups, controlling for age.

Following the observation of statistically significant associations between the UF and TOP-J Informant, we conducted an exploratory follow-up ANCOVA to test whether the relationship between UF and informant-rated judgment differed by diagnostic group, controlling for age. To assess the relationship between structural connectivity and TOP-J scores, each UF ROI-to-ROI connection was entered as a predictor of TOP-J scores in separate regression models, controlling for age. Because this analysis was exploratory, it was not corrected for multiple comparisons.

Statistical analyses were conducted in SPSS software (version 29.0) and R/RStudio (version 2025.05.0 + 496).

## Results

### Participant characteristics

Participants selected for the study were part of a larger sample (*n* = 100). Seven participants had incomplete data across cognitive and neuroimaging measures and therefore were excluded from analysis. Thus, the complete dataset for all analyses was 93 participants between the ages of 60 and 90 years (*M* = 74.83; *SD* = 7.55).

Informants (*n* = 93) ranged in age from 32 to 92 years (*M* = 67.38; SD = 14.34) and were predominantly female (65.6%) with no self-reported history of memory problems. Informants of participants included spouses/partners (44.1%), children (24.7%), friends (23.7%), other relatives (5.4%), parents (1.1%), and professional caregivers (1.1%). All informants had known their respective study participants for at least 2 years (range 2–83 years, *M* = 42.31, *SD* = 17.89).

Table 1 provides participant characteristics. The MCI group was significantly older than the CU group, but there were no differences in sex or educational attainment between groups. As expected, the MCI group was observed to have significantly lower average MoCA scores than CU. In addition, the SCD group was rated as being more vulnerable to financial exploitation on the SVS than the CU group, but not the MCI group. No group differences emerged for the STS.

**Table 1:** Participants characteristics.

	Overall sample (*n* = 93)	CU (*n* = 30)	SCD (*n* = 33)	MCI (*n* = 30)	*p*
Age	74.83 (7.55)	73.57 (1.22)	72.49 (1.13)	78.43 (1.51)	*0.004* (CU < MCI)
Education (years)	15.88 (2.42)	16.10 (0.44)	15.79 (0.40)	15.73 (0.48)	*0.821*
% Female	74.19%	73.33%	81.82%	66.67%	*0.395*
MoCA	25.66 (2.84)	27.30 (0.30)	26.73 (0.35)	22.87 (0.47)	*<0.001* (CU > MCI)
SVS	5.61 (6.05)	3.30 (0.61)	7.06 (1.27)	6.50 (1.12)	*0.030* (CU < SCD)
STS	25.02 (3.42)	25.27 (0.50)	25.33 (0.54)	24.43 (0.79)	*0.523*
A-IADL-Q-SV	62.69 (7.31)	66.83 (3.37)	63.47 (6.28)	59.22 (8.26)	*<0.001* (CU > MCI)

CU, Cognitively Unimpaired; SCD, Subjective Cognitive Decline; MCI, Mild Cognitive Impairment; SVS, Social Vulnerability Scale; STS, Susceptibility to Scams Scale; A-IADL-Q-SV, Amsterdam Instrumental Activities of Daily Living – Short Version. Italic values indicate *p*-values.

A subset of informants (*n* = 6) provided open-ended informant responses describing instances in which participants encountered or engaged with scam attempts. Commonly reported attempts included computer or phone-based fraud. One informant shared:

“*She bought bitcoin from someone on Facebook. It was a distant relative; their Facebook got scammed so it wasn’t really them. She also fell for a romance scam and tried to open a checking account for a person.*”

Others described incidents such as:

“*She got a call from a caller about her credit card balance, and she was falling for their responses to clear it up. Also, a scam to get money out of her bank.*”

“*Phone scam about breach in computer. Allowed scammer to access [her] bank account but stopped and closed accounts when she realized it was a scam.*”

“*Someone wanted him to go to the bank and take out some money. It almost worked.*”

### TOP-J performance across diagnostic groups

The TOP-J and TOP-J Informant were significantly correlated (*r* = −0.249, *p* < 0.05).

We note that the two scales are scored in opposite directions; therefore, a negative correlation indicates agreement. Both TOP-J and TOP-J Informant showed significant diagnostic group differences in the expected directions. There was a statistically significant difference in TOP-J total scores across diagnostic groups as determined by a Kruskal-Wallis *H* test (*H*(2) = 7.829, *p* = 0.020). A pairwise post-hoc test with Bonferroni correction revealed that TOP-J performance was significantly better in the CU group than in the MCI group (*H*(2) = 18.950, *p* = 0.019). Although the SCD group mean TOP-J score was numerically between the CU and MCI group means, it did not significantly differ between the CU (*H*(2) = 13.021, *p* = 0.147) and MCI groups, (*H*(2) = 5.929, *p* > 0.999) Table 2).

**Table 2:** Descriptive statistics and nonparametric analyses on TOP-J total scores for all groups.

	CU *M (SD)*	SCD *M (SD)*	MCI *M (SD)*	Kruskal Wallis *H*	*p*
TOP-J	37.33 (2.77)	35.55 (3.90)	34.40 (4.38)	7.829*	*0.019* (CU > MCI)
TOP-J informant	0.67 (1.18)	3.42 (3.75)	5.30 (6.52)	14.877**	0.*004* (CU < SCD) *0.001* (CU < MCI)

CU, Cognitively Unimpaired; SCD, Subjective Cognitive Decline; MCI, Mild Cognitive Impairment.

*p < 0.05, **p < 0.01. Italic values indicate *p*-values.

Kruskal-Wallis *H* tests revealed a statistically significant difference in TOP-J Informant scores across groups (*H*(2) = 14.877, *p* < 0.001). Pairwise post-hoc analysis with Bonferroni correction demonstrated that the CU group was reported to have significantly better judgment ability than both the SCD (*H*(2) = 20.962, *p* = 0.004) and MCI groups (*H*(2) = 23.183, *p* < 0.001) on the TOP-J Informant. There were no significant differences between SCD and MCI groups (*H*(2) = 2.221, *p* > 0.999).

### TOP-J performance and scam susceptibility

As shown in Table 3, both the TOP-J and TOP-J Informant were associated with responses to the scam engagement question. In contrast, the Social Vulnerability Scale was associated with the TOP-J Informant, but not with the TOP-J. The Susceptibility to Scams Scale was positively associated with the TOP-J but not TOP-J Informant.

**Table 3:** Spearman correlations between scam vulnerability and TOP-J scores in the overall sample.

	Scam engagement question	Social vulnerability scale	Susceptibility to scams scale
TOP-J	0.281*	−0.101	0.243*
TOP-J informant	−0.490**	0.673**	−0.154

*p < 0.05, **p < 0.01.

### TOP-J performance and uncinate fasciculus integrity

Four separate linear regressions were conducted to examine whether FA in the left and right UF predicted TOP-J performance, controlling for age, in the overall group. Age was entered in the first block and FA values were added in the second block.

The TOP-J showed no significant associations with FA values in the left tract (*F*(2, 91) = 0.31, *p* = 0.738, *R*^2^ = 0.007) or right tract (*F*(2, 91) = 0.35, *p* = 0.706, *R*^2^ = 0.008). Table 4 displays the results of the stepwise regressions. For the TOP-J Informant, the model including age and left FA_UF_ significantly predicted scores (*F*(2, 91) = 3.63, *p* = 0.030, *R*^2^ = 0.074). The left UF FA contributed significantly to the model (*p* = 0.018), and this association remained significant after correcting for multiple comparisons (adjusted *a* = 0.025). The corresponding model using the right FA_UF_ was not significant (*F*(2, 91) = 2.78, *p* = 0.067, *R*^2^ = 0.058) and the addition of the right UF (*p* = 0.044) was not significant relative to the adjusted alpha value. Because the TOP-J Informant is scored such that higher values reflect poorer judgment, this association with UF integrity indicates that greater integrity is linked to worse informant-perceived judgment.

**Table 4:** Stepwise linear regressions of age and UF integrity predicting TOP-J performance.

	*B*	*SE*	*β*	*t*	*p*
TOP-J informant					
Age	0.061	0.063	0.098	0.966	0.337
Left UF	77.518	32.035	0.254	2.420	0.018**
Age	0.064	0.064	0.103	1.012	0.314
Right UF	65.200	31.848	0.209	2.047	0.044*
TOP-J					
Age	−0.042	0.054	−0.081	−0.773	0.442
Left UF	4.932	27.516	0.019	0.179	0.858
Age	−0.042	0.054	−0.082	−0.785	0.435
Right UF	9.434	27.106	0.036	0.348	0.729

*p < 0.05, **p < 0.025.

There were no significant differences in left (*F*(2, 89) = 0.01, *p* = 0.986) or right (*F*(2, 89) = 0.10, *p* = 0.905) FA_UF_ among diagnostic groups. Estimated marginal means of left FA_UF_ were approximately equivalent across CU (*M* = 0.277, *SE* = 0.003), SCD (*M* = 0.277, *SE* = 0.003), and MCI groups (*M* = 0.276, *SE* = 0.003). A similar pattern emerged between CU, (*M* = 0.282, *SE* = 0.003), SCD, (*M* = 0.281, *SE* = 0.003), and MCI (*M* = 0.280, *SE* = 0.003) groups in right FA_UF_.

To determine whether the association between the left and right FA_UF_ and informant-rated judgment varied by diagnostic group, we conducted an ANCOVA including a FA_UF_ × diagnostic group interaction term, controlling for age. The interaction terms for the left (*F*(2, 93) = 1.72, *p* = 0.185) and right FA_UF_ (*F*(2, 93) = 1.19, *p* = 0.311) were not significant, indicating that the relationship between FA_UF_ and TOP-J Informant scores did not differ across diagnostic groups.

### TOP-J performance and structural connectivity via the UF

As an additional exploratory analysis, we investigated whether judgment was associated with structural connectivity between temporal and frontal regions. Overall, better judgment (as measured by TOP-J informant scores) predicted stronger frontal and frontal-temporal structural connectivity in select portions of the right UF. Specifically, connectivity between right BA10p and right BA11l (frontal regions) significantly predicted TOP-J informant scores (*F*(2, 89) = 5.97, *p* = 0.01) such that greater structural connectivity between these two regions was associated with better judgment on the TOP-J informant questionnaire (Figure 2). Additionally, connectivity between the right BA10p (frontal region) and right anterior superior temporal gyrus (*F*(2, 89) = 5.91, *p* = 0.01) significantly predicted TOP-J informant scores, such that increased temporal-frontal connectivity was associated with better judgment (Figure 2). None of the right UF connections were significant predictors of the TOP-J. There were no associations between left UF connectivity and TOP-J or TOP-J informant scores across the sample.

Lastly, we examined whether similar associations between informant-reported judgment and white matter integrity extended to other white matter pathways implicated in age-related changes and related cognitive processes such as memory recall (cingulum bundle, Clark et al., 2022; fornix, Senova et al., 2020), language functioning (superior longitudinal fasciculus, Madhavan et al., 2014), and motivation (basal ganglia corticostriatal and thalamic radiation; Haber and Calzavara, 2009). After controlling for age, no significant associations emerged in these analyses. Regression results for exploratory analyses are available in Supplementary Tables 1–2.

## Discussion

Despite the relevance of sound judgment for maintaining independence in aging, this crucial ability remains understudied in older adults without dementia. Although judgment is often included in neuropsychological evaluations of older adults, it is often assessed using proxy measures of executive functioning, such as the Comprehension and Similarities subtests of the Wechsler Adult Intelligence Scale-III (Wechsler, 1997; Wechsler and Scale, 2008) and the Wisconsin Card Sorting Test (Heaton and Staff, 1993), rather than with tools designed to target the complex, multidimensional nature of everyday judgment (Rabin et al., 2008; Faria et al., 2015). Few studies have examined judgment directly using ecologically relevant measures such as the Test of Practical Judgment (TOP-J; Rabin et al., 2008). Moreover, there is a need to take a comprehensive approach to understanding judgment in relation to both real-world outcomes, such as scam susceptibility, and its underlying brain correlates. This knowledge gap is important to fill as structural brain changes have been found to precede observable cognitive decline (Smith, 2012) and contribute to subtle deficits in everyday decision-making.

### Summary of findings

In the current study, we found that both objective (TOP-J) and informant-reported measures of judgment (TOP-J Informant) were significantly associated with item of informant-reported scam engagement in older adults with and without cognitive impairment. We also found that white matter connectivity was associated with decision-making in the context of aging and cognitive impairment. These findings highlight the value of assessing judgment abilities because they can offer quick and practical means of identifying older individuals with compromised judgment consistent with increased vulnerability to scams and fraud.

### Practical judgment across cognitive status

Older adults with and without cognitive impairment showed differences in practical judgment ability consistent with meaningful consequences for real-world functioning. This is consistent with prior work: older adults have been reported to show declines in judgment ability even in the absence of clinically defined cognitive impairment, placing them at risk for poor financial decisions, falling victim to fraud, and substantial financial loss (Rabin et al., 2022; Ueno et al., 2021), and these declines may be exaggerated by subjective or objective cognitive impairment. In our study, the MCI group showed worse judgment than the CU group, based on both objective and informant-reported measures.

Informant-reported judgment scores additionally differentiated SCD from CU participants, suggesting that informant observations may be especially sensitive to earlier differences. The discrepancy between objective scores and informant reports may reflect differences in the nature of what each assessment captures. Informant reports may give valuable insight into ecologically salient capacities and daily functioning that are difficult to assess within a clinical or laboratory setting (Rabin et al., 2022). Informants may also have a longitudinal perspective on a cognitive trajectory that is not available to clinicians. And while objective tests such as the TOP-J were designed to reflect real-life scenarios, these demands may not be easily captured in structured, vignette-based tasks. Informant reporting may therefore offer unique insight into subtle prodromal changes in cognition and judgment. This aligns with prior studies which have shown that informant reports may be more accurate than self-report measures for assessing current cognitive functioning and predicting future cognitive decline (Valech et al., 2015; Numbers et al., 2023), supporting their use alongside standardized performance-based tests.

### Practical judgment and scam vulnerability

Judgment was associated with informant-reported scam vulnerability in the current study. Better objective judgment on the TOP-J was associated with a reduced likelihood of falling (or nearly falling) for scams. Likewise, participants rated as having better judgment by informants (on the TOP-J Informant) were perceived by their informants to be less gullible and more resistant to exploitation. These results suggest that informant-reported and objective tests of judgment offer complementary insights into vulnerability to financial exploitation and highlight the benefits of multi-source evaluation of decision-making capacity.

The SCD group exhibited numerically worse scores on susceptibility and vulnerability measures relative to CU and MCI groups. Although differences in scam susceptibility were not statistically significant, individuals with SCD scored significantly higher than the CU group on informant-reported social vulnerability. This finding suggests that the risk of exploitation may be elevated in older adults with SCD and warrants further investigation in larger samples of this population. This pattern may reflect an inverted U-shaped relationship between cognitive status and fraud vulnerability. Individuals with SCD may maintain enough independence to engage in complex decisions without supervision while experiencing subtle deficits in awareness or judgment, increasing their risk relative to cognitively unimpaired individuals and those with more severe impairment who are less likely to be exposed.

### Practical judgment and white matter correlates

Several studies have explored functional neuroimaging correlates of decision-making (Reiter et al., 2017; Poudel et al., 2017; Labutina et al., 2024) and (moral) judgment (Garrigan et al., 2016; Han, 2017) using performance-based tests. However, there is a lack of research on the brain correlates of practical judgment. Prior work on the uncinate fasciculus (UF) has shown that its microstructural white matter integrity is linked to emotional and motivational processes such as reward-driven processing (Park et al., 2021) and risk-taking behavior (Linke et al., 2013). In the present study, white matter integrity in the left UF predicted informant-reported practical judgment, after controlling for age, suggesting a possible neurobiological correlate of real-world judgment.

Our exploratory region-to-region connectivity analyses revealed that increased connectivity strength among right temporal-frontal and frontal–frontal regions was significantly associated with better informant-reported judgment in the overall sample. Prior studies have uncovered connections between structural and functional brain networks and decision-making (Chen et al., 2019), the importance of frontal connectivity in coordinating behavioral responses (Lamichhane and Dhamala, 2015) and the role of frontal-temporal connectivity for evaluating rewards and guiding choices in healthy adults (Neubert et al., 2015). Whole-brain structural connectivity has also been associated with major cognitive domains (e.g., information processing speed, visuospatial reasoning, and crystallized ability) in healthy older adults (Wiseman et al., 2018). Our findings further extend this work by identifying tract-to-region associations within the UF and linking white matter connectivity to real-world judgment as rated by informants. This finding highlights the potential for informant-based tools to reveal brain-behavior relationships that may not be captured by self or performance-based assessments and suggests that connectivity measures may serve as useful indicators of judgment impairment.

In our overall sample, worse informant-reported judgment was associated with increased FA, indicating greater microstructural integrity in the UF. This pattern was counterintuitive and did not align with studies reporting reduced FA in cognitively impaired groups (Lee et al., 2009; Sexton et al., 2011). Other studies have sometimes noted increased FA values in preclinical populations (Kaļva et al., 2023) and heightened tract-specific connectivity with age (Chakraborty et al., 2025). And at least one published study reported a similar, seemingly paradoxical direct relationship between cognitive performance and FA (Serra et al., 2010). We acknowledge that the negative association of judgment and FA we observed may be spurious, although it remained statistically significant after correction for multiple comparisons. As noted elsewhere, significant obstacles can arise in the interpretation of diffusion-derived findings from older adults and pathological patients due to age-related changes in the brain generally and white matter specifically. Further, some of these changes may exacerbate the known challenges of tract segregation in the context of crossing fibers (Douaud et al., 2011; Gullett et al., 2020).

### Limitations and future directions

Results should be interpreted within the context of study limitations. While the imaging sample size was relatively large compared to other published cross-sectional neuroimaging studies, future work should aim to replicate these findings with a larger sample, and generalization to other racial and ethnic groups would benefit from samples of greater diversity. Additionally, future work would benefit from the inclusion of additional imaging metrics (e.g., functional connectivity) for a more comprehensive assessment. Informant reports may also not be an accurate reflection of judgment for every participant, as reports may vary depending on the informant-participant relationship or degree of familiarity (Hackett et al., 2020). Although several informant and self-report measures of scam susceptibility and fraud vulnerability were included, future studies should explore whether judgment impairments correlate with encountering or engaging with scams. To do so effectively, a more comprehensive measure of scam susceptibility that can capture both the nuance and context of scamming events is needed. Lastly, the cross-sectional nature of the study design limits causal inference between our variables of interest. Future work should enroll older individuals in studies of both cross-sectional and longitudinal design to gain a better picture of differences and changes in judgment, scam susceptibility, and structural connectivity.

### Conclusion

Practical judgment is a critical, yet understudied, construct in aging research that has the potential to capture meaningful real-world vulnerabilities and identify individuals at risk for adverse outcomes such as scam engagement and financial exploitation. Our results emphasize the importance of including multiple sources for judgment assessment to gain a comprehensive understanding of functional risk in older adulthood. This study provides novel, preliminary evidence linking real-world judgment to structural connectivity in the UF, revealing potential structural markers of impaired judgment in older adults without dementia. As judgment may become impaired in non-diagnosed, independently living older adults, it is crucial to continue to uncover its behavioral and brain correlates to inform timely identification of deficits and targeted interventions aimed at preventing exploitation and safeguarding independence.

## Data Availability

The raw data supporting the conclusions of this article will be made available by the authors without undue reservation.

## Footnotes

1 The TOP-J is available upon request: TOPJrequest@gmail.com.
