# Supplementary material for: Practical judgment in aging: examining behavioral vulnerabilities and neurobiological correlates
Source: Front Psychol. 2026 Mar 3;17:1709372. doi: 10.3389/fpsyg.2026.1709372 (PMC12992289; doi:10.3389/fpsyg.2026.1709372)
Supplement: Supplementary file 1 [file Supplementary_file_1.pdf]

Supplementary Table 1. Exploratory age-adjusted associations between white matter integrity (FA) and TOP-J Informant scores

Side	Predictor	*B*	*SE*	*β*	*t*	*p*
L	Age	0.061	0.064	0.098	0.953	0.343
L	Cingulum	44.932	26.866	0.173	1.672	0.098
R	Age	0.056	0.066	0.091	0.855	0.395
R	Cingulum	30.220	24.856	0.129	1.216	0.227
L	Age	0.067	0.064	0.108	1.048	0.297
L	Superior Longitudinal Fasciculus	44.277	27.704	0.164	1.598	0.113
R	Age	0.078	0.064	0.125	1.206	0.231
R	Superior Longitudinal Fasciculus	33.608	27.621	0.126	1.217	0.227
L	Age	0.061	0.065	0.098	0.938	0.351
L	Corticostriatal Tract	32.851	25.490	0.135	1.289	0.201
R	Age	0.068	0.064	0.109	1.059	0.292
R	Corticostriatal Tract	45.245	26.159	0.177	1.730	0.087
L	Age	0.062	0.066	0.099	0.934	0.353
L	Thalamic Radiation	19.312	24.681	0.083	0.782	0.436
R	Age	0.067	0.066	.107	1.011	0.315
R	Thalamic Radiation	-7.843	28.718	-0.029	-0.273	0.785
L	Age	0.076	0.069	0.119	1.095	0.277
L	Fornix	1.753	21.476	0.009	0.082	0.935
R	Age	-0.007	0.056	-0.013	-0.119	0.905
R	Fornix	15.981	11.071	0.153	1.444	0.152

Supplementary Table 2. Exploratory age-adjusted associations between bilateral region to region connectivity within the UF and TOP-J Informant scores

**Right UF Region to Region Connectivity**	*B*	*SE*	*p*
BA 10 to BA 11	-17.671	7.216	0.016
BA 10 to Anterior Insula	-10.785	13.594	0.429
BA 10 to Piriform Cortex	-13.903	19.432	0.476
BA 10 to Superior Temporal Gyrus	-33.918	13.955	0.017
BA 10 to Temporal Pole	-8.363	5.709	0.146
BA 11 to Anterior Insula	-1.271	7.238	0.861
BA 11 to Piriform Cortex	-0.900	10.896	0.934
BA 11 to Temporal Pole	-5.355	4.994	0.286
BA 11 to Superior Temporal Gyrus	-17.168	12.132	0.161
Anterior Insula to Piriform Cortex	-1.354	2.064	0.513
Anterior Insula to Superior Temporal Gyrus	-5.882	11.373	0.606
Anterior Insula to Temporal Pole	-5.197	6.014	0.389
Superior Temporal Gyrus to Piriform Cortex	-1.126	12.348	0.927
Superior Temporal Gyrus to Temporal Pole	-13.591	7.152	0.061
Piriform Cortex to Temporal Pole	-2.222	3.753	0.555
**Left UF Region to Region Connectivity**	*B*	*SE*	*p*
BA 10 to BA 11	-14.999	16.899	0.377
BA 10 to Anterior Insula	-4.633	9.032	0.609
BA 10 to Piriform Cortex	9.254	53.094	0.862
BA 10 to Superior Temporal Gyrus	-6.918	18.285	0.706
BA 10 to Temporal Pole	-0.985	7.948	0.901
BA 11 to Anterior Insula	-65.249	82.538	0.431
BA 11 to Piriform Cortex	23.773	35.934	0.509
BA 11 to Temporal Pole	-0.778	8.451	0.926
BA 11 Superior Temporal Gyrus	4.9060	13.067	0.708
Anterior Insula to Piriform Cortex	-10.714	12.405	0.390
Anterior Insula to Superior Temporal Gyrus	-3.716	25.283	0.883
Anterior Insula to Temporal Pole	-9.642	9.488	0.312
Superior Temporal Gyrus to Temporal Pole	-0.733	1.558	0.639
Piriform Cortex to Superior Temporal Gyrus	0.826	30.598	0.978
Piriform Cortex to Temporal Pole	0.0697	3.844	0.985
